# Evidence for Widespread Genomic Methylation in the Migratory Locust, *Locusta migratoria* (Orthoptera: Acrididae)

**DOI:** 10.1371/journal.pone.0028167

**Published:** 2011-12-05

**Authors:** Katie L. Robinson, Donya Tohidi-Esfahani, Nathan Lo, Stephen J. Simpson, Gregory A. Sword

**Affiliations:** 1 School of Biological Sciences, The University of Sydney, Sydney, Australia; 2 Australian National University College of Medicine, Biology and Environment, Australian National University, Canberra, Australia; 3 Department of Entomology, Texas A&M University, College Station, Texas, United States of America; University of Otago, New Zealand

## Abstract

The importance of DNA methylation in mammalian and plant systems is well established. In recent years there has been renewed interest in DNA methylation in insects. Accumulating evidence, both from mammals and insects, points towards an emerging role for DNA methylation in the regulation of phenotypic plasticity. The migratory locust (*Locusta migratoria*) is a model organism for the study of phenotypic plasticity. Despite this, there is little information available about the degree to which the genome is methylated in this species and genes encoding methylation machinery have not been previously identified. We therefore undertook an initial investigation to establish the presence of a functional DNA methylation system in *L. migratoria*. We found that the migratory locust possesses genes that putatively encode methylation machinery (DNA methyltransferases and a methyl-binding domain protein) and exhibits genomic methylation, some of which appears to be localised to repetitive regions of the genome. We have also identified a distinct group of genes within the *L. migratoria* genome that appear to have been historically methylated and show some possible functional differentiation. These results will facilitate more detailed research into the functional significance of DNA methylation in locusts.

## Introduction

DNA methylation is a taxonomically widespread epigenetic marker to which many non-exclusive functions have been attributed, among them genomic imprinting and the regulation of phenotypic plasticity [1]–[5]. It was originally considered doubtful that DNA methylation was biologically significant in insects, based upon the results of initial studies using *Drosophila melanogaster* adults [6]. Subsequent work confirmed the existence of methylcytosine in dipteran insects [7], but showed that it is present in very small amounts and, at least in *D. melanogaster*, limited to early embryonic developmental stages [8]. Similarly low levels of DNA methylation were found in the coleopteran *Tribolium castaneum* [9], [10]. More recently, however, a growing body of research has indicated that in a number of insect species DNA methylation is both present at appreciable levels and regulates diverse and important biological processes. In honeybees (*Apis mellifera*) it is involved in caste differentiation [4] and learning and memory [11]. In mealybugs (*Planococcus citri*) it is thought to be responsible for genomic imprinting [2] and in the peach aphid (*Myzus persicae*) has been associated with pesticide resistance [12]–[14].

In contrast to the high levels of global methylation characteristic of vertebrates, it appears that DNA methylation in insects has a mosaic distribution throughout the genome, occurs predominantly within genes, and does not play a large role in the suppression of repetitive DNA [9], [15]. Accordingly, the proportion of the genome that is methylated is typically reported to be much smaller in insects [1], [16]–[18]. However, there are some examples of insect species with relatively higher levels of DNA methylation [19] and in one case this appears to be associated with the methylation of repetitive DNA [20]. Overall, DNA methylation in insects is still not well characterised, with the potential diversity in its functional roles remaining largely unexplored. It is also unclear how much variation exists across taxa in the amount and distribution of DNA methylation within the genome and its significance to organismal biology.

The migratory locust (*Locusta migratoria*) is an economically important species of insect with a globally widespread distribution [21]. Individuals display a striking polyphenism, exhibiting either a solitarious or gregarious phase phenotype depending upon maternal epigenetic cues [22]–[24] and the population density that they have experienced [25], [26]. The expression of these density-dependent phenotypic changes in behaviour and other traits can play a causal role in locust swarm formation [27]–[29]. Phase polyphenism is generally accepted to have arisen independently multiple times within locusts [30] and it is unclear to what extent the underlying molecular mechanisms are conserved between species. Until very recently, there was only one report of DNA methylation in locusts, with estimates of methylcytosine content obtained using a rudimentary paper chromatography technique [31]. This is somewhat surprising given the significance of these species as models for the study of phenotypic plasticity. The presence of DNA methylation and DNA methyltransferase encoding genes has now been confirmed in the desert locust *Schistocerca gregaria* [32], but the function of DNA methylation in locusts is yet to be determined. Here we provide novel evidence that the genome of the migratory locust is both methylated and contains genes encoding methylation machinery orthologs. We show that a fraction of the genome enriched in methyl-CpG sites contains repetitive sequences, including a SINE retrotransposon known to be differentially expressed between isolated and crowded locusts [33]. We also demonstrate that a subset of locust genes display evidence of historical methylation within their protein encoding sequences. The degree of both current and historical genomic methylation indicated by our results to be present in the migratory locust suggests the possible role of DNA methylation in regulating locust phenotypic plasticity as a promising avenue of future study.

## Results

### Methylation-specific restriction enzyme assays

**Figure 1:**
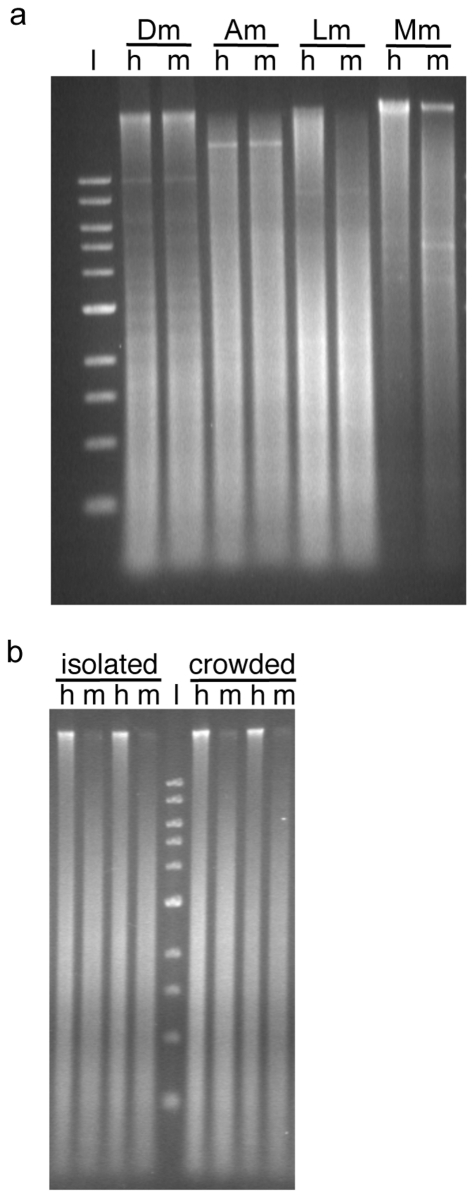
Extant genome-wide methylation. (a) DNA extracted from adult head tissue of *D. melanogaster* (Dm), *A. mellifera* (Am) and *L. migratoria* (Lm) and 3T3 fibroblasts of *M. musculus* (Mm) was digested separately with *Hpa*II and *Msp*I. Approximately 1 µg of DNA per digest was loaded onto the gel. L, NEB 1 KB ladder; h, *Hpa*II; m, *Msp*I. (b) *Hpa*II/*Msp*I digested *L. migratoria* egg DNA. Tissue for DNA extraction was pooled from four individuals per egg pod. Each egg pod was obtained from a different mother. Approximately 1 µg of DNA per digest was loaded onto the gel. Lanes 1–4, isolated pods 1 and 2 respectively; lanes 6–9, crowded pods 1 and 2 respectively. Data presented here are representative of the ten replicates performed per treatment.

Digestion of both *L. migratoria* egg and adult head DNA with *Hpa*II (5′-CCGG-3′), which is sensitive to methylation of the internal cytosine, revealed a pronounced high molecular weight fraction that largely disappeared in the methylation-insensitive *Msp*I (5′-CCGG-3′) digest, clearly indicating the presence of methylated CpG sites within this DNA (Figure 1a, b). In contrast, no distinction was evident between the *Hpa*II and *Msp*I digests of either *D. melanogaster*, the adult genome of which contains virtually no methylcytosine, or the honeybee, which possesses a functional CpG methylation system, but relatively low levels of genomic methylation (Figure 1a). A much larger proportion of the *Mus musculus* (mouse) DNA was observed to be resistant to *Hpa*II digestion than the insect DNA, consistent with the relatively high levels of CpG methylation observed in mammals. The majority of the high molecular weight fraction was cleaved by *Msp*I, and in this species, unlike the insect representatives, there was also a noticeable reduction in the average fragment length obtained from the *Msp*I digestion relative to the *Hpa*II digest. No obvious differences in CpG methylation were detected between the genomic DNA of eggs from isolated and crowded locusts using this method (Figure 1b). The restriction pattern of genomic DNA from crowded adult head tissue was comparable to that of egg tissue (Figure 1a, b).

### Cloning and sequencing of methylcytosine enriched genomic DNA

The high molecular weight fraction of *L. migratoria* genomic DNA seen following digestion with *Hpa*II was not present for the *Msp*I digest, which indicates that this DNA is enriched in methylated CpG sites. In an effort to further characterise this portion of the genome, we purified the DNA and then cloned and sequenced a number of fragments. Omitting bacterial contaminants, a total of 49 clones were obtained, representing 40 unique sequences (Table 1). One third of these clones (31%) were found to have homology to repetitive DNA, with the *L. migratoria* retroelement, LmI the most highly represented sequence. A number of clones (18%) showed significant similarity (E value = 4e^−178^ – 2e^−4^) to unannotated *L. migratoria* and *S. gregaria* ESTs, the identity of which could not be determined using BLAST searches. Approximately 51% of the clones could not be reliably identified using BLAST searches and are likely to represent uncharacterised regions of the genome.

**Table 1:** Identity of cloned DNA sequences derived from methyl-CpG enriched portion of the *L. migratoria* genome.

Sequence type	Sequence class	Number of clones (%)
Repetitive DNA		15 (31%)
	*LmI retrotransposon*	*8 (16%)*
	*Other transposon*	*3 (6%)*
	*Tandem repeat*	*2 (4%)*
	*Other*	*2 (4%)*
Unannotated locust EST		9 (18%)
Unknown sequence		25 (51%)

### Observed and expected CpG ratios

**Figure 2:**
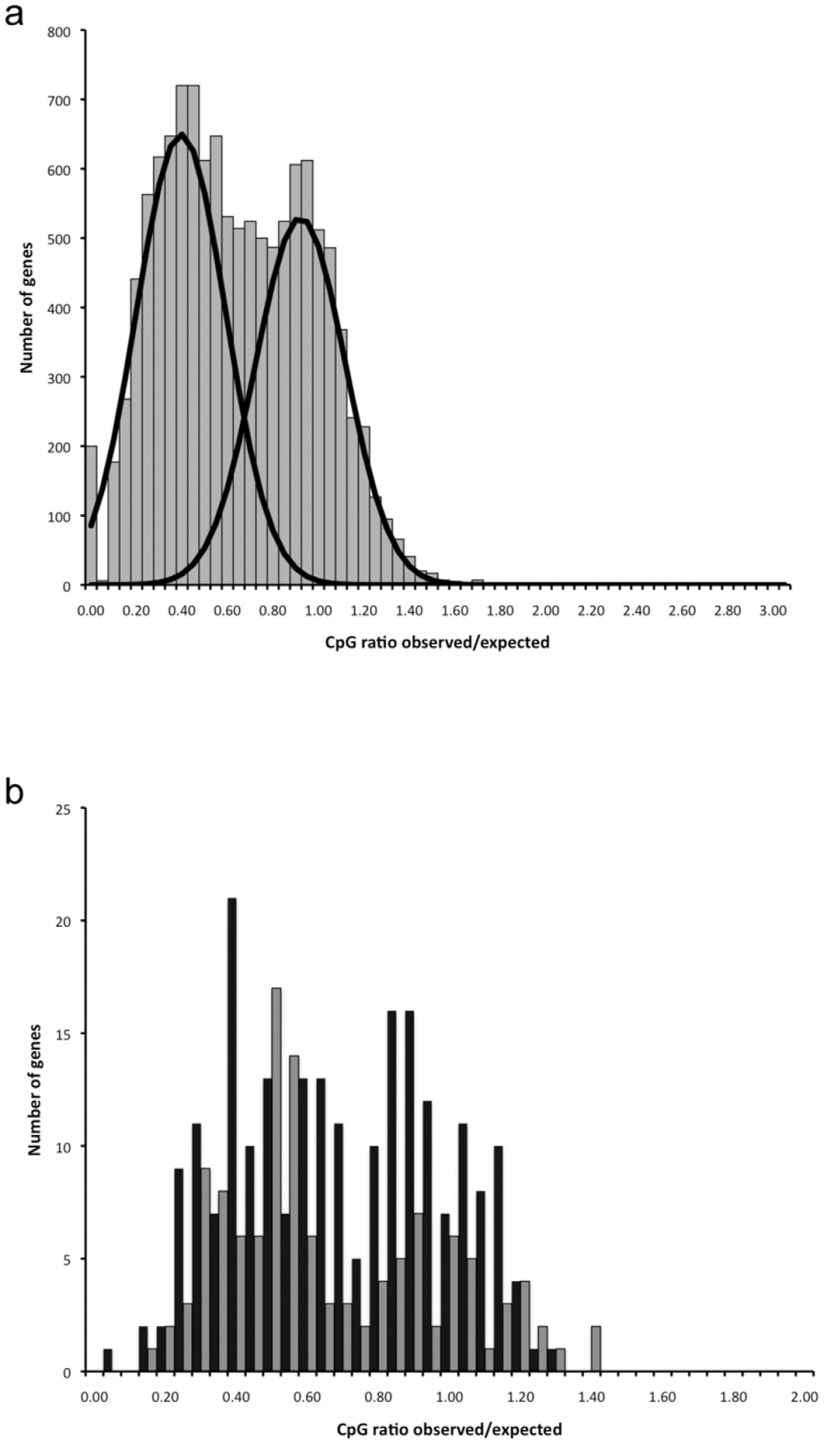
Historic genome-wide methylation. (a) Actual calculated CpG*_O/E_* gene frequency values for the entire unigene dataset are presented as a histogram to which predicted normal distributions have been fitted (File S1). (b) Calculated CpG*_O/E_* gene frequency values for a subset of unigenes known to be differentially expressed between isolated and crowded locusts; black bars = genes upregulated in isolated locusts, grey bars = genes upregulated in crowded locusts.

Due to the hypermutability of methylcytosine to thymine, sequences that are highly methylated in the germline are predicted to undergo CpG depletion over evolutionary time [34], [35]. The ratio of observed to expected CpG sites (CpG*_O/E_*) in a sequence can therefore be used to make inferences about the level of historic DNA methylation that occurred in the germline. As no genome sequence information was available for *L. migratoria* at the time of the present study, analysis of CpG sites was performed using a data set of 12,148 unigenes downloaded from a publicly available EST database [36]. The distribution of unigene CpG*_O/E_* ratios is clearly bimodal (Figure 2; unimodal distribution rejected, p<0.05), with two peaks centred at approximately 0.4 and 0.9. This suggests that the *L. migratoria* genome contains two distinct populations of genes, those that have an observed CpG frequency close to that expected by chance and those in which the CpG content is much lower. A similar distribution of CpG*_O/E_* ratios was observed when 343 unigenes previously demonstrated to be differentially expressed between isolated and crowded *L. migratoria* [37] were examined separately (Figure 2b). This pattern was observed regardless of whether genes were more highly expressed in isolated or crowded locusts. Whereas CpG*_O/E_* shows a pronounced bimodal distribution, GpC*_O/E_* and all of the other dinucleotide pairs display unimodal gene frequencies for the complete unigene dataset (Figure S1). This implies that there has been little bias exerted upon the CpG*_O/E_* distribution by the GC content of the genes and that the bimodality of CpG*_O/E_* is likely to be attributable to the historic presence of methylcytosine in the germline. The existence of the low CpG*_O/E_* population of unigenes suggests that widespread historical gene body methylation has occurred in a CpG context in this species, and that this extends to some of the genes involved in regulating phase polyphenism. Although there is strong evidence of CpG depletion and historic methylation within the *L. migratoria* genome, caution should be exercised when interpreting precisely to what extent this occurs, as it is possible that the distribution of CpG*_O/E_* ratios has been influenced by positional biases in the distribution of ESTs within trancripts and methylated CpG sites within gene bodies [9]. Gene Ontology (GO) functional enrichment analyses (biological process) of a subset of 2,482 unigenes for which annotation data was available showed that transport, carbohydrate metabolism, proteolysis and peptidolysis, chitin metabolism and muscle development genes were overrepresented in the high CpG*_O/E_* group (p<0.05), whereas metabolism genes were significantly enriched in the low CpG*_O/E_* class of genes (Table 2).

**Table 2:** Results of functional enrichment analysis.

CpG*_O/E_* class	GO term	EASE score	Benjamini
low	*Metabolism*	*<0.01*	*<0.01*
	Protein transport	<0.01	0.04
	Intracellular protein transport	<0.01	0.09
	Biosynthesis	<0.01	0.15
	Electron transport	<0.01	0.23
high	*Transport*	*<0.01*	*<0.01*
	*Carbohydrate metabolism*	*<0.01*	*<0.01*
	*Proteolysis and peptidolysis*	*<0.01*	*<0.01*
	*Chitin metabolism*	*<0.01*	*<0.01*
	*Muscle development*	*<0.01*	*0.03*

For both low and high CpG*_O/E_* gene populations, the five most overrepresented GO terms are presented. The EASE score is a modified Fisher exact probability p-value, the Benjamini value is the raw EASE score corrected for multiple comparisons using the Benjamini- Hochberg false discovery rate method [72]. Significantly overrepresented functional categories (p<0.05) are shown in italics.

### Identification of L. migratoria methylation machinery

**Figure 3:**
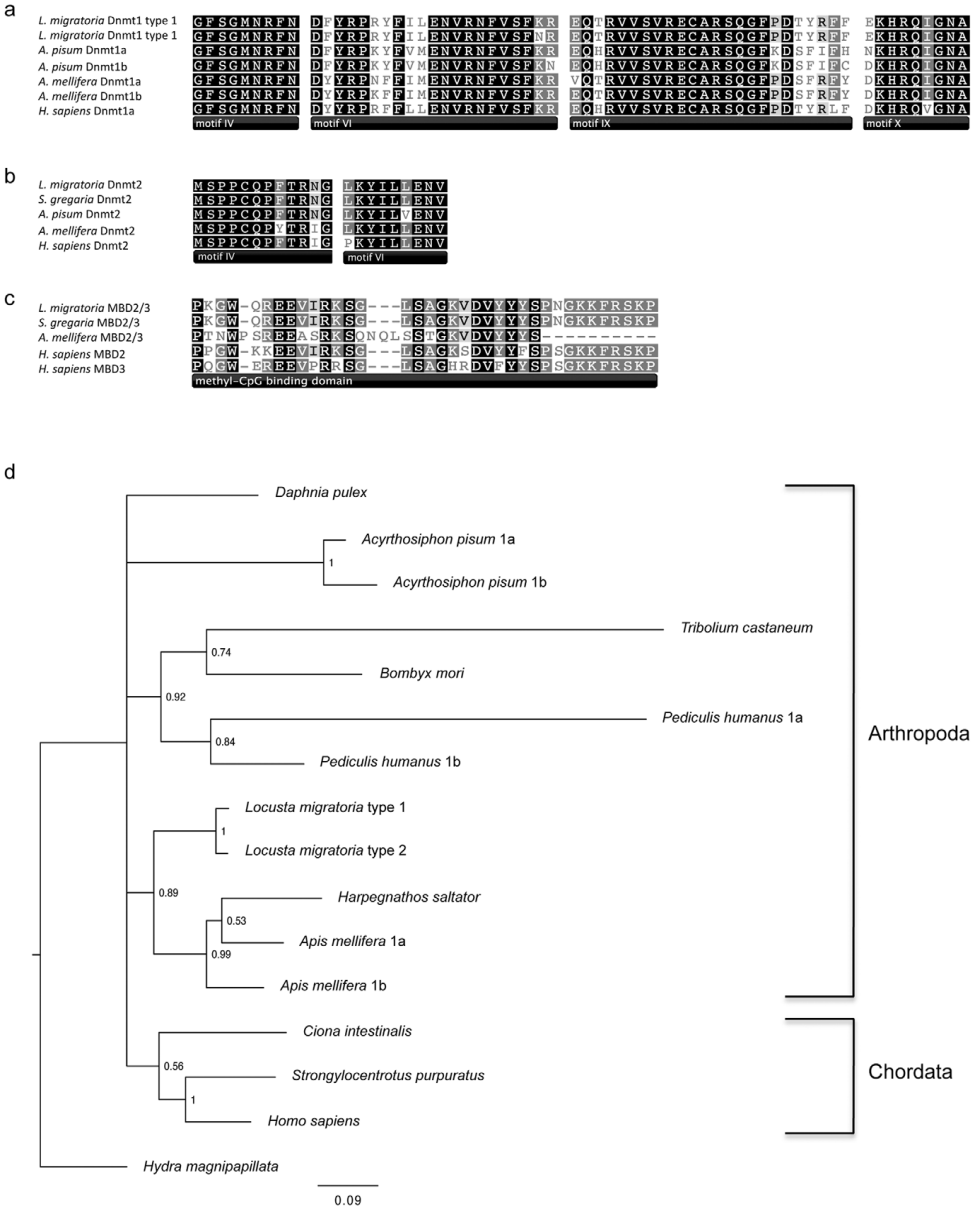
Methylation machinery of *L. migratoria*. ClustalW alignment of (a) Dnmt1, (b) Dnmt2 and (c) MBD2/3 partial sequences. For clarity, only regions with homology to conserved motifs are presented. Sequences from a larger selection of species were used for phylogenetic analyses, but for simplicity, only a subset of these is shown here. The reported *S. gregaria* Dnmt1 sequence could not be aligned with the other Dnmt1 sequences presented here. Completely conserved residues are shaded black; 80–99% identical residues are dark grey; 60–79% identical residues are light grey; less than 60% identical residues are white. Alignments were constructed using MEGA4 [74] and formatted with Geneious Pro 4.8.5 [76]. Accession numbers are as follows: *Acyrthosiphon pisum* Dnmt1a, XP_001942687.1; *A. pisum* Dnmt1b, XP_001942632.1; *Apis mellifera* Dmnt1a, NP_001164522.1; *A. mellifera* Dnmt1b, XP_001122269; *Bombyx mori* Dnmt1, BAD67189.1; *Ciona intestinalis* Dnmt1, XP_002122948.1; Daphnia pulex Dnmt1, EFX80183.1; *Harpegnathos saltator* Dnmt1, EFN76367.1; *Homo sapiens* Dnmt1, NP_001124295.1; *Hydra magnipapillata* Dnmt1, XP_002155714.1; *L. migratoria* Dnmt1 type 1, FR850040; *L. migratoria* Dnmt1 type 2, FR850041; *Pediculus humanus* Dnmt1a, XP_002432160.1; *P. humanus* Dnmt1b, XP_002431878.1; *Strongylocentrotus purpuratus* Dnmt1, XP_780273.1; *T. castaneum* Dnmt1, XP_001814230.1; *A. pisum* Dnmt2, XP_001949338; *A. mellifera* Dnmt2, XP_393991; *H. sapiens* Dnmt2, 1G55_A; *L. migratoria* Dnmt2, FR850042; *A. mellifera* MBD2/3, XP_392422.2; *L. migratoria* Dnmt1 MBD2/3, FR850043; *H. sapiens* MBD2, NP_003918.1; *H. sapiens* MBD3, NP_003917.1. (d) Bayesian phylogenetic analysis of animal Dnmt1 sequences. In constructing the tree 339 amino acid sites were used. Posterior probabilities greater than 0.5 and the expected number of substitutions per site (scale bar) are indicated. Two distinct *Pediculis humanus* Dnmt1 sequences were present in GenBank. As their isoform type was not specified, they were arbitrarily named Dnmt1a and Dnmt1b. Accession numbers are listed above.

DNA sequence data was obtained for two of the three animal methyltransferases, the DNA methyltransferase (Dnmt1) and the tRNA methyltransferase (Dnmt2) as well as the invertebrate methyl-CpG binding domain protein (MBD2/3). The corresponding amino acid sequences contained conserved motifs characteristic of these proteins (Figure 3). Two discrete *L. migratoria Dnmt1* cDNA variants, designated *Dnmt1* type 1 and type 2 and sharing 96.9% identity at the amino acid level (340/351 sites) were identified from two clones. Bayesian analysis of Dnmt1 sequences grouped all of the arthropod representatives together (Figure 3). Dnmt1a and Dnmt1b sequences were not found to cluster according to gene class; for insect species in which two *Dnmt1* genes have been identified, greatest similarity was found between the two Dnmt1 variants within a species.

## Discussion

The present study provides the first evidence that the genome of *L. migratoria* contains genes encoding DNA methylation machinery, and demonstrates using two independent lines of evidence that the genome is methylated to a considerable degree. Differences in the pattern of restriction fragments observed following digestion of *L. migratoria* DNA with *Hpa*II/*Msp*I, which is indicative of the level of CpG methylation, were not as pronounced as for *M. musculus*, which exemplifies the high level of genomic methylation characteristic of mammals. The amount of cytosine methylation present for *L. migratoria* appears to be higher than that of *D. melanogaster* and *A. mellifera*, the latter of which has a functional DNA methylation system [18], although it is possible that this is partly due to variation in the distribution of *Hpa*II/*Msp*I sites within the genome as well as differences in the amount of CpG methylation. The proportion of methylcytosine in the genome of another locust, *S. gregaria*, has recently been estimated to be 1.3–1.9% in different nervous and endocrine tissues [32]. In agreement with the *L. migratoria* digest results, these values are intermediate to those calculated for *D. melanogaster* embryos and *A. mellifera* (0.1% and 0.3% respectively) [9] and mammals (4–6%) [38]. Digestion of *L. migratoria* DNA was similar to that reported for the stick insect *Medauroidea extradentata* [20] and the mole cricket *Gryllotalpa fossor* [39] and is reminiscent of that seen in higher deuterostome invertebrates [40]. Together with the finding that the cabbage moth (*Mamestra brassicae*) possesses relatively high levels of DNA methylation [19], these results highlight the variability in the amount of methylation present among insect genomes, the majority of which have been shown to be sparsely methylated to date.

Genome-wide analyses of methylation have been conducted at base pair resolution in two insects, the honeybee and the silk moth (*Bombyx mori*). In these species it has been shown that DNA methylation is localised primarily to gene bodies and at least in *B. mori*, sequences encoding small RNAs, whereas transposable elements are hypomethylated [9], [16], [41]. It appears that while there is evidence of gene body methylation in *L. migratoria*, CpG methylation is not limited to the regions described for *A. mellifera* and *B. mori*. As for the phasmid *M. extradentata* [20], substantial methylation of repetitive sequences is also likely to be present, as indicated by the 31% of clones from the methyl-CpG enriched portion of the *L. migratoria* genome found to contain repetitive DNA. Recent studies have indicated that DNA methylation is involved in stabilising gene expression [35] and regulating the production of alternative transcripts [42]. Despite these insights, the role of DNA methylation and its functional conservation throughout different taxa is still not fully understood. It is possible that in the Orthoptera and Phasmatodea DNA methylation plays a part in controlling repetitive elements in addition to influencing gene expression. The proportion of repetitive DNA in the *L. migratoria* genome has been estimated to be 30% [43], ten times more than of *A. mellifera* [44]. The most abundant sequence represented in the cloned methyl-CpG enriched *L. migratoria* DNA corresponds to a previously characterised SINE retrotransposon, LmI [45], which alone accounts for approximately 2% of the genome. Interestingly, this element has been found to show differential expression in the nervous tissue of isolated and crowded *L. migratoria* [46] and is a source of small RNAs [47]. The methylation of repetitive DNA in the Orthoptera and Phasmatodea could be related to the very large genome size of these species [48]–[50], although other aspects of organismal biology are also likely to be important in determining levels of genomic methylation [17]. Indeed, repetitive DNA is estimated to comprise 45% of the sparsely methylated *B. mori* genome [16], [51].

Perhaps unsurprisingly, no clear difference was observed when comparing the *Hpa*II/*Msp*I digestion patterns of solitarious phase locusts to those of gregarious ones. The lower sensitivity of this method, which was unable to detect the biologically significant CpG methylation present in the *A. mellifera* genome, means that only relatively dramatic shifts in genome-wide methylation are likely to be identifiable. Finer scale mapping may reveal biologically meaningful heterogeneity in the methylomes of solitarious versus gregarious locusts. For example, bisulphite sequencing has already been used to investigate differences in gene methylation between phenotypic variants in both the honeybee and the pea aphid [1], [4], [42]. The evidence of historical methylation shown here for some genes known to be differentially expressed between isolated and crowded locusts suggests that this will be an worthwhile area of investigation.

Complementary DNA sequencing confirmed that the *L. migratoria* genome contains orthologs of the invertebrate methyl-binding protein MBD2/3 [52] and at least two of the three animal Dnmts; the maintenance methyltransferase, Dnmt1 [53] and the tRNA methyltransferase, Dnmt2 [54]. It is possible that Dnmt3, a proposed *de novo* methyltransferase [53], is also present. However, work towards identifying this gene has been problematic thus far. It is interesting to note that *Dnmt3* does not appear to occur in either the *S. gregaria* [32] or *L.migratoria* [36] EST databases. Further research is required to clarify whether this gene is conserved in locusts as it is in the Hymenoptera [18], [55]–[59] and the aphid *A. pisum* [1], or like in the representatives of other insect orders (*T. castaneum* [55], *B. mori* [16], *D. melanogaster* [56], [57], *Anopheles* gambiae [7] and *Pediculis humanus* [58]), appears to have been lost.

It is now becoming apparent that there is considerable interspecific variation in the number of Dnmt1 proteins that insects possess [59]. In some lineages Dnmt1 appears to have been lost entirely, while other species have up to three variants. Whereas humans possess two Dnmt1 isoforms encoded by a single gene [60], in insects multiple Dnmt1 isoforms are encoded by separate genes. The degree of polymorphism observed between the two *L. migratoria* Dnmt1 variants suggests that they correspond to different genes. The tree topology observed for the insect Dnmt1 proteins is in agreement with observed sequence differences and the previous observation that multiple copies of the gene appear to have arisen independently in several different insect lineages.

The *L. migratoria* genome displays evidence of widespread historical gene methylation in the germline, with unigenes falling into two distinct populations; low CpG*_O/E_* genes which show evidence of historical methylation and high CpG*_O/E_* genes which are likely to have been hypomethylated in the evolutionary past. In other species it appears that these historical estimates are likely to be a good predictor of current levels of gene methylation [35], [61]. Thus, it may be that in *L. migratoria* many of the low CpG*_O/E_* genes undergo present-day methylation, at least in the germline.

Analyses of genome-wide methylation patterns in a diverse range of organisms have revealed a parabolic relationship between levels of gene body methylation and transcription, with both silent and highly expressed genes typically exhibiting little or no methylation and genes with intermediate levels of expression the most strongly methylated [9]. It has therefore been suggested that a major function of DNA methylation is to prevent aberrant transcripts arising from basic biological process genes that have broad, moderate expression [35]. In accordance with this, metabolism was the most overrepresented term in the low CpG*_O/E_* group of *L. migratoria* unigenes, similar to *A. mellifera* [62]. It remains to be seen if DNA methylation is involved in the phase-specific regulation of metabolism genes, which a previous study has identified as being differentially expressed between isolated and crowded fifth instar locusts [37], consistent with the differing metabolic requirements of solitarious and gregarious individuals [26], [63].

The high CpG*_O/E_* group of *L. migratoria* unigenes was functionally enriched with five gene categories, the expression of three of which (muscle development, proteolysis and peptidolysis and carbohydrate metabolism) varies according to locust phenotype [37]. It is important to note that the predicted hypomethylation of these genes in the past does not preclude the involvement of DNA methylation in their regulation; genes that lack methylation in the germline and are therefore not subject to CpG depletion over evolutionary time may still be methylated in a particular environmental or developmental context.

### Conclusions

The ecology, ethology and physiology of locust phase polyphenism are becoming well understood. The lack of locust genome sequence data available at present and the size of the genome, which is approximately 30 times larger than that of *D. melanogaster* [64], have posed specific challenges to understanding the molecular processes underlying locust phenotypic plasticity. Considerable progress has been made in identifying genes differentially expressed between the solitarious and gregarious phases [37], [65], but the regulatory mechanisms governing these differences remain elusive. Our results provide clear evidence of genomic methylation in *L. migratoria*, with methylation occurring in both repetitive and protein-encoding regions of the genome. They also show that metabolic process genes, which are known to be differentially expressed in isolated versus crowded locusts [37], are the most proportionally overrepresented genes in a population of protein-encoding sequences that are likely to have been historically methylated. DNA methylation has been implicated in phenotypic plasticity and genomic imprinting in a diverse range of species, including some insects [1]–[5]. Given that locust phase change is a form of phenotypic plasticity that in some species involves the epigenetic transfer of phase state from parent to offspring, investigating the possible role of DNA methylation in regulating phase change in locusts represents a promising new area of research in this economically significant group of insects.

## Materials and Methods

### Locust culture and experimental material

A *L. migratoria* culture was established as described earlier [66]. Isolated and crowded individuals were reared essentially as specified for *Schistocerca gregaria* [67], [68]. For both isolated and crowded treatments, eggs were harvested from egg pods on the ninth day after oviposition, when locusts were in the final stages of embryonic development. Isolated eggs were derived from mothers that were the offspring of crowded parents but were raised in isolation from approximately two days prior to hatching and were mated with males reared in the same manner.

### Methylation-specific restriction enzyme assays

DNA was extracted from *L. migratoria*, *D. melanogaster* and *A. mellifera* adult head tissue and *L. migratoria* egg tissue using Genomic Tips (Qiagen). *M. musculus* genomic DNA was obtained commercially (New England Biolabs) and was derived from the embryonic fibroblast cell line NIH 3T3. Restriction digests with *Hpa*II and *Msp*I (New England Biolabs) were carried out for 4 hr at 37°C with 2 µg of genomic DNA and 10 U of enzyme in a final volume of 50 µl. Digested DNA was separated on 0.8% agarose/TAE gel and visualised using ethidium bromide staining.

### Cloning and sequencing of methylcytosine enriched genomic DNA

Genomic DNA from the eggs of ten crowd-reared mothers was pooled for this analysis. The high molecular weight genomic DNA that appeared as a discrete band following *Hpa*II digestion and agarose electrophoresis was excised from the gel and purified with the Wizard SV Gel and PCR Clean-Up System (Promega). DNA was further purified and concentrated by ethanol precipitation, using standard methods. Approximately 0.35 µg of purified DNA was then digested with 20 U *Taq*I (Roche) for 4 hr at 65°C in a final volume of 50 µl. Digested DNA was ethanol precipitated and used to perform a ligation reaction with pUC19 DNA that had been linearised with NarI (New England Biolabs) and treated with Antarctic phosphatase (New England Biolabs). Ligation was carried out with T4 DNA ligase (New England Biolabs) at 16°C overnight. Recombinant DNA was used to transform chemically competent *Escherichia coli* TOP10 cells (Invitrogen) and positive transformants were identified using colony PCR. Sequencing was performed by Macrogen, Inc. (Korea) and clones were identified by performing NCBI BLAST [69] searches for homologous sequences (blastn algorithm; default parameters). Search terms were initially restricted to insect EST sequences. For clones that could not be identified, search terms were expanded to the entire nucleotide collection. The identity of insect EST sequences that produced top hits with the *L. migratoria* clones was determined using the blastx algorithm (default parameters).

### Observed and expected CpG ratios

Analysis was performed using a data set of 12,148 unigenes downloaded from a publicly available database, LocustDB; http://locustdb.genomics.org.cn/ [36]. The database is derived from seven different cDNA libraries, which were constructed using the whole bodies of crowded locusts and head, hind leg and midgut tissue from isolated and crowded individuals. The unigenes in the dataset have a mean length of 598 bp (range = 101–4,278 bp). CpG*_O/E_* ratios were calculated as described previously [1], [62], [70], incorporating a correction for the presence of Ns in the sequence data (File S1). Bimodality of the CpG*_O/E_* ratios was assessed using the statistical program NOCOM to test whether the gene frequency distribution differed significantly from a unimodal distribution [62], [71]. A subset of 2,482 unigenes for which pre-existing GO biological process annotation data was available in LocustDB was examined further to investigate whether there was any proportional overrepresentation of functional categories in low versus high CpG*_O/E_* gene populations. Analyses were performed essentially as described elsewhere [62], with the stand-alone DAVID application EASE used to perform statistical calculations [72]. Functional enrichment was assessed by comparing the number of occurrences of each GO term in low and high CpG*_O/E_* gene groups to a background list of all annotated unigenes.

### Identification of Dnmt and MBD sequences

Degenerate primers (Table 3) were designed based upon alignments of published gene orthologs from other species, either manually or using the program CODEHOP [73]. PCR fragments were amplified from cDNA template, cloned and sequenced using standard methods. RNA was extracted from locust tissue using Trizol reagent (Invitrogen). cDNA was synthesised using the SuperScript III First-Strand Synthesis System (Invitrogen). PCR products were cloned using the TOPO TA Cloning Kit for Sequencing (Invitrogen) and sequencing was performed by Macrogen, Inc. (Korea). DNA sequences were translated and then aligned with orthologous proteins using the default ClustalW function of MEGA4 [74]. For Bayesian analyses, tree topologies were estimated from the dataset with MrBayes 3.1 [75]. The model of protein evolution was selected automatically within MrBayes (prset aamodelpr = mixed). Two independent analyses were run for 1 million generations, each starting with different random trees with four chains, with sampling every 100th generation. The first 3000 sampled generations were considered the burn-in and discarded. Posterior probabilities were determined by constructing a 50% majority-rule tree of the 7000 sampled post-burn-in trees. The mixed model sampled the WAG matrix with a posterior probability of 1.00. In effect, this is the same as running the analysis using a fixed WAG matrix.

**Table 3:** Primers used in this study.

Gene	Primer	Sequence (5′-3′)
*Dnmt1*	forward	TGTGCGGAGGACCACCNTGYCARG
	reverse	CCCAGTGGTGGTGGCACNGCRTTNC
*Dnmt2*	forward	ATGAGTCCTCCNTGYCARCC
	reverse	TTCAAATCCTTTNACRTTYTC
*MBD*	forward	CCGAAAGGATGGCAAAGAGAAGAAG
	reverse	TGAGGTTTGCTGCGAAACTTCTTCC

## Supporting Information

Figure S1 **Observed to expected ratios for all dinucleotide combinations.** The number of genes is shown on the y-axis. For clarity *O/E* values greater than 3.00, where present, are not represented; omitting this data did not affect the shape of any of the distributions.
(TIF)

File S1 **Calculation and analysis of observed and expected CpG ratios.**
(DOC)
