# Supplementary figures and images for: Evidence for Widespread Genomic Methylation in the Migratory Locust, Locusta migratoria (Orthoptera: Acrididae)
Source: PLoS One. 2011 Dec 5;6(12):e28167. doi: 10.1371/journal.pone.0028167 (PMC3230617; doi:10.1371/journal.pone.0028167)

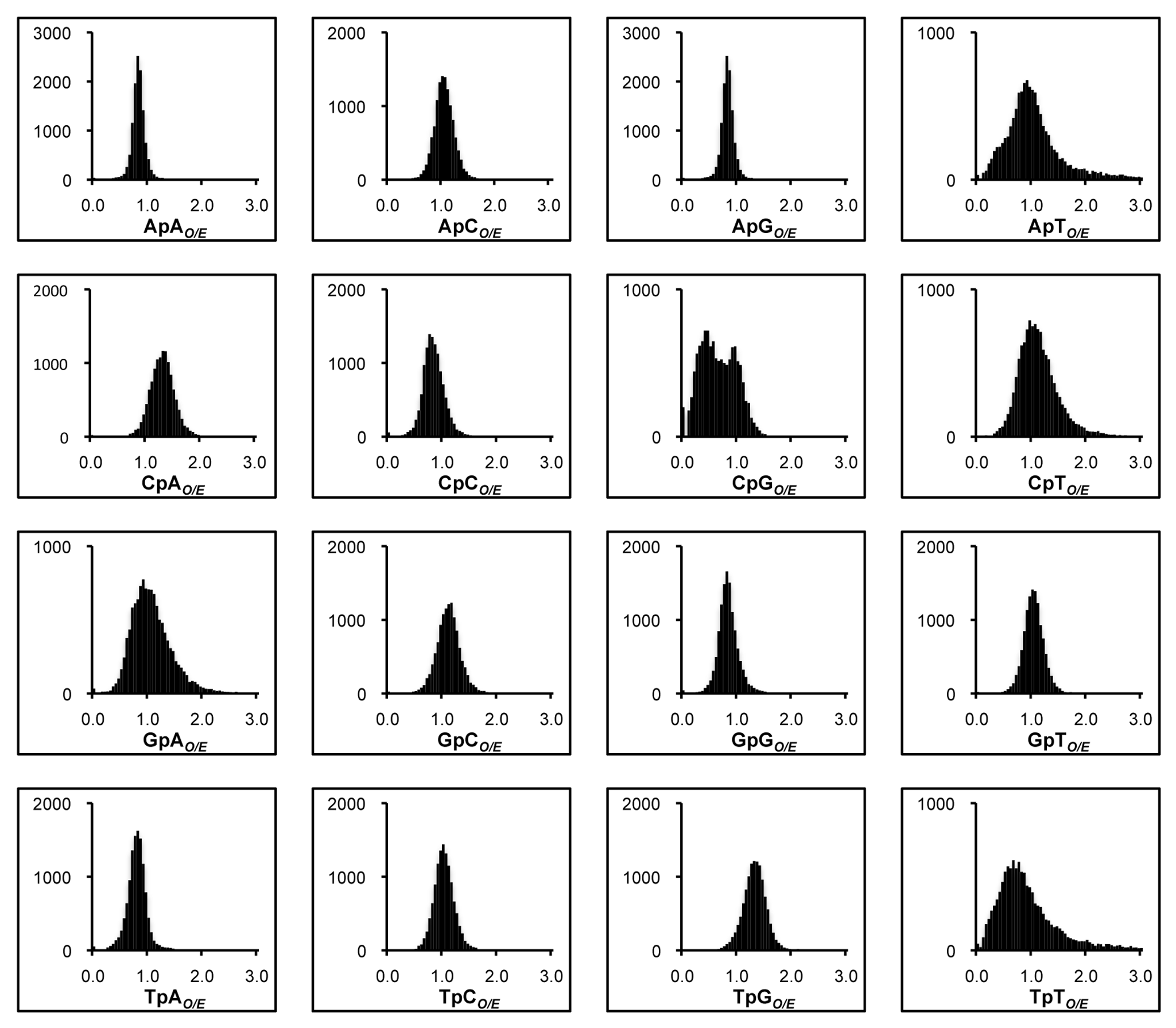

Supplement: Figure S1 — Observed to expected ratios for all dinucleotide combinations. The number of genes is shown on the y-axis. For clarity O/E values greater than 3.00, where present, are not represented; omitting this data did not affect the shape of any of the distributions. (TIF) [file pone.0028167.s001.tif]
