# Supplementary material for: Evidence for Widespread Genomic Methylation in the Migratory Locust, Locusta migratoria (Orthoptera: Acrididae)
Source: PLoS One. 2011 Dec 5;6(12):e28167. doi: 10.1371/journal.pone.0028167 (PMC3230617; doi:10.1371/journal.pone.0028167)
Supplement: File S1 — Calculation and analysis of observed and expected CpG ratios. (DOC) [file pone.0028167.s002.doc]

**Supplementary Information**

**Observed and expected CpG ratios**

CpG*_O/E_* ratios were calculated for each unigene in Microsoft Excel using the formula CpG*_O/E_* = P_CpG_ /P_C_*P_G_, where P_CpG_ represents the observed CpG frequency and P_C_ and P_G_ are the proportion of C and G nucleotides in the unigene, respectively. Measures of P_CpG_ were obtained by dividing the observed number of CpG dinucleotides in a unigene by the number of dinucleotide positions. In sequences without Ns, the number of dinucleotide positions is equal to the sequence length minus one. For sequences containing Ns, the effective number of dinucleotides was determined by taking the sum of the dinucleotide positions in each run of known sequence interspersed by one or more N base pairs. Observed frequencies of C and G nucleotides were determined individually for each unigene. The effective sequence length, defined here as the total length of the unigene minus the number of N base pairs was used for these calculations. The possible effect of sequence quality upon the results was assessed by performing a duplicate analysis that excluded from the dataset all sequences less than 300bp in length and/or containing 30 or more mononucleotide repeats. Removal of these unigenes was found to have a negligible influence upon the CpG*_O/E_* gene frequency distribution (data not shown). The complete dataset was thus used for subsequent analyses. To confirm that the CpG*_O/E_* values were independent of unigene GC content, GpC*_O/E_* was calculated as above, along with the observed/expected ratios for all other dinucleotide combinations.

Normal curves were fitted to each unimodal component of the bimodal CpG*_O/E_* gene frequency distribution data using the normal distribution function of Excel and the means and standard deviations estimated for each component by NOCOM. The normal frequency values were transformed to match the original data by dividing each individual frequency by the sum of all frequencies for that component and then multiplying by the number of genes predicted by NOCOM to be included in each component. The intercept of the two normal curves was determined algebraically. Genes with a CpG*_O/E_* value below the intercept were assigned to the low CpG*_O/E_* gene class and those with a CpG*_O/E_* ratio above the intercept were placed in the high CpG*_O/E_* class.
